# Digital Precision vs. Visual Perception: A Comparative Evaluation of Shade Selection Reliability Using Intraoral Scanning and Conventional Shade Guides

**DOI:** 10.7759/cureus.97890

**Published:** 2025-11-26

**Authors:** Morupuri Sunitha, Thota K Mohan, Gottumukkala Vineela, Goli Mounika Reddy, Amulya T, Kurlagunda Himaja, Seema Gupta

**Affiliations:** 1 Department of Prosthodontics, Sibar Institute of Dental Sciences, Guntur, IND; 2 Department of Conservative Dentistry and Endodontics, Government Dental College and Hospital, Hyderabad, IND; 3 Department of Pedodontics and Preventive Dentistry, Chadalawada Krishnamurthy Sekhar Theja Dental College and Hospital, Tirupati, IND; 4 Department of Periodontics, National Dental Care Clinics, Hyderabad, IND; 5 Department of Orthodontics, Kothiwal Dental College and Research Centre, Moradabad, IND

**Keywords:** guide, matching, scanner, tooth shade, visual

## Abstract

Introduction

Shade selection remains a critical yet challenging aspect of restorative and esthetic dentistry, particularly in the anterior region, where even minor discrepancies can compromise outcomes and patient satisfaction. This study aimed to evaluate the intra-method repeatability and inter-method agreement of shade selection using the conventional Vita Classical shade guide (Vita Zahnfabrik, Bad Säckingen, Germany) versus the 3Shape Trios 4 intraoral scanner (3Shape A/S, Copenhagen, Denmark) in a clinical setting.

Materials and methods

A prospective within-subject comparative study was conducted on 40 dental students aged 20-30 years with healthy unrestored maxillary central incisors. Each participant underwent shade assessment of both central incisors using both methods in three separate sessions under standardized illumination in a color-controlled operatory. Visual selection was performed by a calibrated prosthodontist using the Vita Classical guide, while digital analysis was carried out using the 3Shape Trios 4 scanner with factory-calibrated software. The teeth were cleaned and isolated prior to measurements, and the procedures were sequenced by blinding between methods. Data were recorded electronically and analyzed for reliability using appropriate statistical tests.

Results

The intra-method repeatability was significantly higher with the digital scanner (Fleiss κ = 0.83, p = 0.001) than with the visual guide (Fleiss κ = 0.25, p = 0.001). Inter-method agreement across all sessions was negligible (Cohen’s κ = 0.03-0.05, p > 0.05), with 95% confidence intervals.

Conclusion

The 3Shape Trios 4 intraoral scanner provided highly reproducible shade data; however, the results differed substantially from those of conventional visual matching. These methods are not interchangeable. A combined approach using digital tools for consistency and visual assessment for clinical correlation is recommended to enhance the accuracy and predictability of esthetic restorations.

## Introduction

Accurate shade selection is a cornerstone of restorative and esthetic dentistry, profoundly impacting the success of prosthetic restorations and patient satisfaction. In the anterior esthetic zone, where subtle color discrepancies are readily noticeable, achieving a seamless blend between natural teeth and restorations demands precision beyond mere technical skill [1]. Conventional visual shade matching, primarily using guides such as the Vita Classical shade guide (Vita Zahnfabrik, Bad Säckingen, Germany), remains the most widespread clinical approach owing to its accessibility and familiarity. However, this method is inherently subjective and is influenced by ambient lighting, operator fatigue, color perception variability among clinicians, and the complex optical properties of teeth, such as regional variations in hue, chroma, value, and translucency [2,3]. These factors contribute to inconsistent outcomes, often necessitating remaking and compromising on laboratory communication.

The advent of digital dentistry has introduced objective alternatives, including spectrophotometers and intraoral scanners with integrated shade-analysis capabilities. Devices such as the 3Shape Trios series capture high-resolution three-dimensional (3D) impressions while simultaneously quantifying tooth color through automated algorithms, reducing human bias, and enabling standardized data transfer to dental technicians [4,5]. Preliminary studies suggest that digital systems offer enhanced repeatability and reduced inter-operator variability compared to visual methods; however, clinical evidence directly comparing their reliability in routine practice remains sparse [6,7]. Moreover, the learning curve associated with repeated assessments and the influence of environmental standardization on both modalities warrant further exploration.

This comparative clinical study was conducted to evaluate the reliability of shade selection using the conventional visual method with the Vita Classical shade guide versus the digital method employing the 3Shape Trios 4 intraoral scanner (3Shape A/S, Copenhagen, Denmark). This study aimed to assess intra- and inter-method repeatability through repeated measurements, thereby determining the potential of digital integration to augment traditional workflows and improve esthetic predictability in restorative dentistry.

## Materials and methods

Study design and setting

This prospective comparative clinical study employed a within-subject design, wherein each patient served as their own control and underwent shade selection via both modalities at multiple time points to assess intra- and inter-method repeatability. This study was conducted at the Sibar Institute of Dental Sciences, Guntur, Andhra Pradesh, India. The study duration spanned four months, from May 2024 to August 2024, encompassing patient recruitment, data collection, and preliminary analysis.

Ethical approval was obtained from the Institutional Ethics Committee of the Sibar Institute of Dental Sciences (Pr.355/IEC/SIBAR/2024), in accordance with the Declaration of Helsinki and the Indian Council of Medical Research guidelines. Written informed consent was obtained from all patients prior to enrolment following a detailed explanation of the study's purpose, procedures, potential risks (minimal, such as temporary discomfort from cheek retraction), benefits (contribution to improved shade matching techniques), and confidentiality measures. The patients were informed of their right to withdraw at any stage without prejudice. The data were anonymized using unique identifiers, and no personal health information was disclosed.

Eligibility criteria

A purposive sampling method was used to recruit 40 dental students, aged 20-30 years. The inclusion criteria were as follows: healthy, unrestored maxillary central incisors with no visible caries, pigmentation, enamel/dentin defects, or orthodontic appliances; absence of habits such as smoking or tobacco use that could alter shade; and willingness to attend scheduled sessions. The exclusion criteria included the presence of any restorations, active caries, extrinsic/intrinsic stains, periodontal disease, enamel hypoplasia, fixed orthodontic appliances, or systemic conditions affecting oral pigmentation (such as fluorosis). The patients underwent a preliminary oral examination by a calibrated prosthodontist to confirm their eligibility. Of the 50 individuals screened, 40 met the inclusion criteria, yielding a recruitment rate of 80%.

Sample size estimation

The sample size required to achieve a power of 80% and a 5% alpha error for the study was 40. The estimation is performed using the following power calculation formula:

\begin{equation} n = \frac{(Z_{\alpha/2} + Z_{\beta})^2}{\kappa_1^2} \end{equation}

where,

n = sample size;

Z_α_ = 1.96 at 95% confidence;

Z_β_ = 0.84 at 80% power; and

κ = observed reliability between intraoral scanner and manual shade guide methods (κ = 0.43 as given by Liberato et al. [6]).

Methodology

Participants were allocated to a single cohort without randomization, as the within-subject design compared the methods directly on the same teeth. Two shade selection methods were used in this study. The conventional visual method utilizes the Vita Classical shade guide, comprising 16 porcelain tabs arranged by hue (A: reddish-brown, B: reddish-yellow, C: gray, D: reddish-gray), chroma, and value. This guide, the gold standard for visual matching, allows for a rapid comparison under controlled illumination.

The digital intraoral scanner method employed a 3Shape Trios 4 intraoral scanner (3Shape A/S, Copenhagen, Denmark) integrated with proprietary shade analysis software. This wireless scanner captures high-resolution 3D intraoral scans (up to 2,000 frames/s) with real-time color mapping using multi-spectral light-emitting diode (LED) illumination, automatically classifying shades against the Vita Classical system via algorithmic interpolation.

All procedures adhered to a strict protocol to ensure reproducibility and minimize bias. Participants were seated in a semi-reclined position in a dedicated operatory with neutral gray walls to reduce metameric effects. Ambient lighting was standardized using a Verivide Color Assessment Cabinet (Just-Normlicht GmbH, Weilheim an der Teck, Germany) providing a daylight simulation (D65, 6500 Kelvin, 1000 lux at tooth level), which was verified daily using a lux meter. Operator fatigue was controlled by limiting sessions to 10 patients per day, with the same calibrated prosthodontist (intra-examiner κ > 0.85 from pilot testing) performing visual assessments.

Prior to each session, the Vita Classical shade guide was calibrated by verifying tab integrity and color stability against a reference spectrophotometer (Vita Easyshade V, Vita Zahnfabrik), ensuring a color difference value of Delta E (ΔE) less than 1.0 according to the International Commission on Illumination Lab (CIE Lab*) color space. The 3Shape Trios 4 intraoral scanner underwent factory-recommended calibration using its integrated white-balance wand and software update (version 2.1.0), followed by a phantom tooth scan to confirm accuracy within 10 μm trueness and shade repeatability (κ > 0.90).

**Figure 1:**
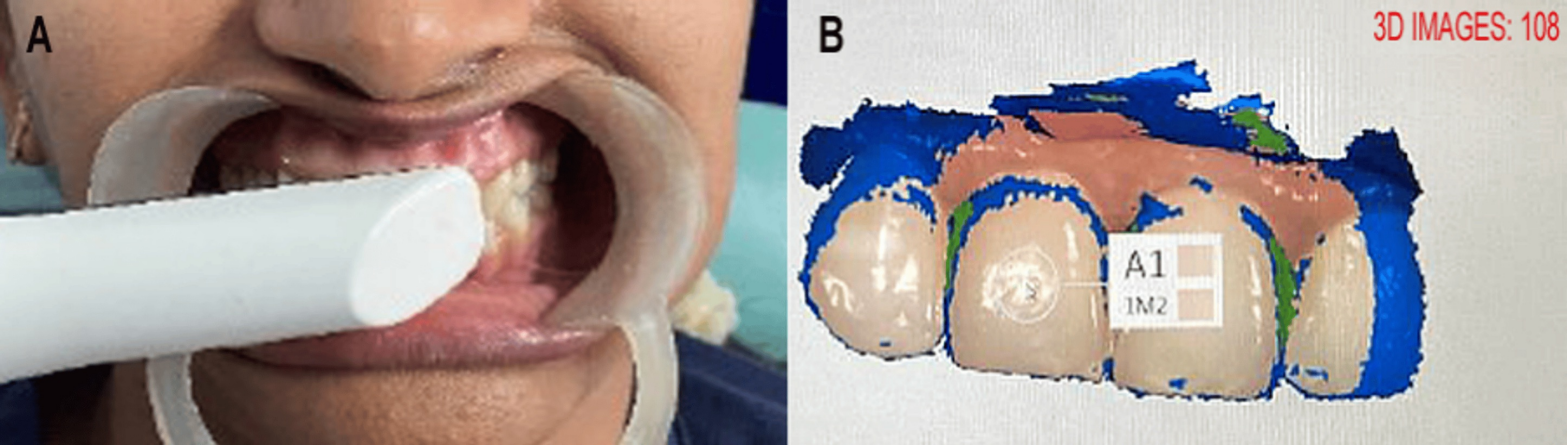
(A) Perception of shade with intraoral 3Shape Trois 4 intraoral scanner. (B) Selection of shade of upper central incisors with software. Original images of the study participant, used with permission.

The procedure was as follows: First, the teeth were gently cleaned using a low-pressure water spray and sterile cotton pellets to remove plaque without desiccation, followed by air drying for five seconds, then placement of cheek retractors (Safari OptraGate, Hager Worldwide, Hickory, NC) and cotton rolls to expose the maxillary central incisors fully, isolating the esthetic zone. For the visual method, the clinician positioned the shade guide tabs parallel to the tooth midline at a 90° angle, 2-3 cm distance, under D65 light, selecting the closest match in <30 seconds to simulate clinical efficiency. Immediately thereafter, the digital method involved a full-arch scan (occlusal to incisal) with the Trios 4 wand held perpendicular to the tooth surface at 5-10 mm, capturing shade data automatically via the software's color engine (Figure 1). The results were exported as shade codes (e.g., A2) and documented in a blinded electronic datasheet. Each method was blinded to the others until post-session data entry. Sessions lasted 10-15 minutes, with no intervention between the methods. Adverse events were monitored; however, no adverse events occurred.

Statistical analysis

Data analysis was performed using Statistical Package for the Social Sciences (version 25.0, IBM Corp., Armonk, NY). The normality of the data was checked using the Shapiro-Wilk test and was found to be normally distributed. The reliability of shade selection within the method (digital intraoral scanning and shade guide) for multiple measurements was analyzed by Fleiss’ kappa, and agreement between the shades matched by the different methods was tested with weighted Cohen’s kappa. The level of significance was set at p < 0.05.

## Results

Reliability analysis revealed poor to slight agreement between the intraoral scanner and the conventional shade guide across all three time points, with all Cohen's kappa values being negligible (κ = 0.03-0.05) and statistically non-significant (p > 0.05). The 95% confidence interval for these agreement scores was zero, further confirming the lack of consistent agreement between the two methods. By contrast, the internal consistency of each method differed substantially (Table 1).

**Table 1:** Inter-method agreement in shade selection between the Vita Classical shade guide at different time points. κ: Cohen’s kappa, Cohen’s κ < 0.20: poor agreement, CI: confidence interval. All p-values > 0.05 indicate non-significant agreement.

Measurements	Cohen's kappa	Standard error	95% CI lower limit	95% CI upper limit	z-value	p-value
First time	0.03	0.072	-0.10	0.16	0.40	0.691
Second time	0.05	0.061	-0.06	0.15	0.61	0.540
Third time	0.05	0.063	-0.06	0.17	0.67	0.502

The shade guide demonstrated fair agreement among repeated measurements (Fleiss κ = 0.25, p = 0.001), whereas the intraoral scanner showed almost perfect agreement (Fleiss κ = 0.83, p = 0.001). While the intraoral scanner is a highly reliable tool for reproducible shade matching, its results are not interchangeable with those obtained from a conventional shade guide. This significant discrepancy indicates that the two methods cannot be used interchangeably in clinical practice, as they likely capture different color parameters or are influenced by different variables (Table 2).

**Table 2:** Intra-method repeatability of shade selection using the Vita Classical shade guide and 3Shape Trios 4 intraoral scanner across three measurements. κ: Fleiss’ kappa, Fleiss’ κ 0.21-0.40: fair, 0.81-1.00: almost perfect, CI: confidence interval. *p = 0.001 denotes highly significant agreement.

Method	Fleiss’ kappa	Standard error	95% CI lower limit	95% CI upper limit	z-value	p-value
Vita Classical shade guide	0.25	0.051	0.16	0.35	5.26	0.001*
3Shape Trios 4 scanner	0.83	0.064	0.71	0.94	14.00	0.001*

## Discussion

The present study provided compelling evidence of the comparative reliability of conventional visual shade selection using the Vita Classical shade guide and digital shade analysis with the 3Shape Trios 4 intraoral scanner in a controlled clinical setting. The key findings revealed a stark contrast between the intra-method repeatability and inter-method agreement. Although the digital scanner demonstrated almost perfect internal consistency, the visual method yielded only fair agreement. However, the inter-method agreement was negligible, indicating that the two approaches are not interchangeable despite mapping to the Vita Classical system.

These results align with emerging literature, highlighting the superior repeatability of digital shade tools. For instance, Tabatabaian et al. [8] conducted a systematic review to evaluate the accuracy and precision of intraoral scanners in shade determination, and included 17 studies. They found that ten articles reported low accuracy and seven articles reported high levels of precision in shade matching with intraoral scanners, attributing this to multi-spectral LED illumination and algorithmic standardization that mitigate human perceptual errors. Similarly, Floriani et al. [9] reported no significant differences in shade determination using a Trios 3Shape intraoral scanner and spectrophotometer; however, their accuracy decreased after aging. Czigola et al. [7] advised the use of both intraoral and classical shade guides for accurate shade determination. Liberato et al. [6] reported the best accuracy of shade determination using the 3Shape Trios intraoral scanner (Fleiss’ kappa value of 0.874). The Vita Classical shade guide method without a light-correcting device showed the poorest reliability (Fleiss kappa value of 0.177). Similar findings were reported by Igiel et al. [3], who concluded that visual shade matching exhibited a high to moderate level of inconsistency for both intra- and inter-rater comparisons. The almost perfect repeatability of Trios 4 in our study supports these observations, as the scanner's AI-enhanced color engine processes real-time data from thousands of frames, minimizing influences such as operator fatigue or ambient metamerism, factors that plagued the visual method, where even a calibrated examiner achieved only fair consistency.

In contrast, poor inter-method agreement underscores the fundamental discrepancies in how shade is captured and interpreted. The Vita Classical guide relies on subjective perceptual matching under standardized D65 lighting, yet regional tooth variations (such as incisal translucency vs. cervical chroma) often lead to holistic rather than segmented assessments [1]. However, digital scanners employ spectral analysis across the tooth surface, interpolating to the nearest Vita tab using proprietary algorithms. This was evident in our data, where scanner outputs frequently deviated by one or more tabs, echoing the findings of Liberato et al. [6], who reported a moderate kappa (0.43) between intraoral scanner and visual methods, but noted systematic biases toward higher chroma in digital readings due to surface gloss capture. Our lower kappa values may stem from stricter environmental controls and a younger cohort with minimal staining, which amplifies subtle optical differences.

Previous studies have yielded mixed results regarding interchangeability. In an investigation conducted by Yoon et al. [10], the mean values of color difference (ΔE) between hues recorded by an intraoral scanner and those measured by a colorimeter surpassed 10. In another study, it was reported that intraoral scanning devices exhibited significant variations when juxtaposed with Easyshade V. Furthermore, these intraoral scanners provided a limited spectrum of color options and documented the highest luminosity levels in comparison to alternative methodologies [11]. Digital methodologies provide superior consistency compared to visual assessment systems. Factors such as the translucency of the enamel, color of the background, intensity of light, and subjective circumstances of the evaluator can affect the tooth shade perceived by human vision. Nevertheless, visual matching continues to be regarded as a benchmark technique because of its ease of use and minimal expense [12].

Our findings reinforce this, suggesting that while digital integration streamlines workflows, facilitating direct CAD/CAM data transfer and reducing remakes, it cannot yet supplement visual verification in esthetic zones. Lee and Kim [13] reported low agreement between visual matching and the scanner method (kappa value of 0.140). They concluded that, although the scanner can estimate tooth color, it should be verified by visual matching. In a literature review by Tabatabaian et al. [14], 249 articles were considered, and it was concluded that digital methods showed higher accuracy and precision than visual methods.

The negligible agreement found in our study has implications for future hybrid protocols. In routine practice, clinicians may employ the scanner for initial objective mapping, followed by visual confirmation to bridge perceptual gaps. This could enhance predictability in prosthetic fabrication, particularly for porcelain-fused-to-metal or zirconia restorations, where ΔE < 2.0 is clinically acceptable [15]. Moreover, the reproducibility of the scanner supports longitudinal monitoring of shade stability post-bleaching or aging, areas that are underexplored in visual methods prone to drift.

Clinical implications

Digitally assisted shade selection offers transformative potential in restorative dentistry by providing consistent, bias-free data that improve technician communication and minimize remakes. In esthetic-driven practices, integrating intraoral scanners, such as Trios 4, can standardize protocols across multi-operator clinics, fostering evidence-based decisions. For complex cases involving translucency or gradients, digital outputs enable layered ceramic builds with precise color zoning. However, until algorithms fully align with human vision, a dual-method approach is prudent: using digital for repeatability and visual for patient-centric validation, ultimately boosting satisfaction and efficiency.

Limitations

Although this study was conducted under rigorously standardized conditions, several limitations should be acknowledged. The restricted sample size and single-center recruitment may limit the generalizability of the findings to wider populations with diverse clinical characteristics. Participants were young adults with healthy, unrestored maxillary central incisors, which do not reflect variations seen in older teeth, extrinsic or intrinsic staining, or restored dentitions. Only the Vita Classical shade guide was used, and other multidimensional shade systems were not evaluated. Despite strict lighting control and examiner calibration, factors such as subtle tooth surface variability, psychovisual bias, and possible observer fatigue may have influenced visual shade selection. Moreover, the controlled operatory environment may not fully represent everyday clinical lighting scenarios. Finally, while repeated measurements improved internal validity, the study did not assess long-term shade stability or clinical restoration outcomes, which should be explored in future research to strengthen external relevance.

## Conclusions

This study demonstrated that the digital method using the 3Shape Trios 4 scanner demonstrated excellent intra-method repeatability, indicating its potential value as a consistent and objective support tool for shade documentation. However, due to the lack of a direct colorimetric accuracy assessment, such as ΔE validation against a spectrophotometer, and the absence of clinical acceptability thresholds or patient-based outcome evaluation, the scanner cannot yet be regarded as clinically superior or interchangeable with conventional visual shade matching. Future research incorporating standardized color difference metrics, independent instrumental comparison, and patient-centered esthetic acceptability data is needed to establish whether high repeatability translates into meaningful clinical improvement.
